# Comment to: ChatGPT: immutable insertion in health research and researchers’ lives

**DOI:** 10.31744/einstein_journal/2024CE1115

**Published:** 2024-09-09

**Authors:** Hinpetch Daungsupawong, Viroj Wiwanitkit

**Affiliations:** 1 Private Academic Consultant Lao People's Democratic Republic Brazil Private Academic Consultant, Phonhong, Lao People's Democratic Republic.; 2 University Centre for Research Development Department of Pharmaceutical Sciences Mohali Punjab India University Centre for Research & Development Department of Pharmaceutical Sciences, Chandigarh University, Mohali, Punjab, India.

Dear Editor,

Vieira et al. brought forward interesting observations in their study "ChatGPT: immutable insertion in health research and researchers’ lives.^(1)^" The article demonstrated how ChatGPT's unchangeable information insertion can transform health research and the lives of researchers. In this sense, artificial intelligence is promising for improving researcher collaboration and streamlining the research process. The writers did an excellent job of highlighting ChatGPT's advantages for healthcare and how it can further medical research.

However, it does not detail the potential drawbacks or moral dilemmas of using this technology. Future studies should investigate these in more detail to ensure that ChatGPT is applied sensibly and morally in the context of health research.

Moreover, regarding potential future developments, it would be fascinating to observe how ChatGPT may be combined with other cutting-edge technologies, such as blockchain, to improve data security and openness in medical research. In addition, ChatGPT's potential to be used in clinical settings to help medical professionals make informed judgments should be investigated. The developments in these fields may substantially impact the precision and speed of medical diagnosis and treatment decisions.

It is critical that scientists continue investigating ChatGPT's potential in diverse facets of health research and deal with any obstacles that might arise. Researchers can ensure that ChatGPT is used efficiently and ethically in the healthcare industry by considering the larger ramifications of this technology and its ethical implications. Lastly, because every ChatGPT prompt and associated output depends on human user input, a human user behavior code is required.^(2)^
